# Neoantimycin F, a *Streptomyces*-Derived Natural Product Induces Mitochondria-Related Apoptotic Death in Human Non-Small Cell Lung Cancer Cells

**DOI:** 10.3389/fphar.2019.01042

**Published:** 2019-09-18

**Authors:** Liyun Liu, Hongrui Zhu, Wei Wu, Yaoyao Shen, Xiao Lin, Ying Wu, Li Liu, Jie Tang, Yongjun Zhou, Fan Sun, Hou-Wen Lin

**Affiliations:** ^1^Research Center for Marine Drugs, State Key Laboratory of Oncogenes and Related Genes, Department of Pharmacy, Ren Ji Hospital, School of Medicine, Shanghai Jiao Tong University, Shanghai, China; ^2^School of Life Sciences and Biopharmaceutical Sciences, Shenyang Pharmaceutical University, Liaoning, China; ^3^College of Pharmacy, Jinan University, Guangzhou, China

**Keywords:** non-small cell lung cancer (NSCLC), neoantimycin F, cell cycle, apoptosis, MMP

## Abstract

*Streptomyces*-derived natural products have been become a major focus of anti-tumor drug discovery studies. Neoantimycin F (NAT-F), was isolated from *Streptomyces conglobatus* by our group. Here, we examined the anti-cancer activities and its underlying molecular mechanisms implicated in NAT-F–induced apoptosis of non–small cell lung cancer (NSCLC) cells. Our results showed that NAT-F exerted excellent growth-inhibitory activity against PC9 and H1299 cells in a concentration-dependent manner. NAT-F–induced cell cycle arrest at S and G_0_/G_1_ phase in PC9 and H1299 cells, respectively. Further investigation revealed that the key proteins (including cyclinD1, cyclinE1, cyclinB1, CDK2, and CDK4) were involved in the cell regulation by NAT-F. Additionally, NAT-F significantly increased the production of reactive oxygen species (ROS), induced DNA damage, nuclear condensation, and cell apoptosis in both cell lines. Moreover, loss of the mitochondrial membrane potential (MMP) was markedly induced by NAT-F. Additional results revealed that NAT-F could up-regulate pro-apoptotic protein Bax and down-regulate anti-apoptotic protein Bcl-2, Mcl-1, and Bcl-x_L_, resulting in cytochrome c release from mitochondria and sequential activation of caspase-9 and -3, as well as the cleavage of poly (ADP-ribose) polymerase. Meanwhile, c-Jun N-terminal kinase (JNK), p38 MAPK (p38), and extracellular signal-regulated kinase (ERK) signaling pathway were also involved in anti-cancer activity of NAT-F in NSCLC cells. Taken together, these findings indicated that NAT-F possessed anti-proliferative effect and induced apoptosis in NSCLC cells *in vitro* and may be conducive to promote the development of novel anti-NSCLC agents.

## Introduction

Lung cancer is one of the leading causes of cancer death worldwide, and non-small cell lung cancer (NSCLC), the most common histologic type, accounts for approximately 75% to 85% of all lung cancers (Aisner and Marshall, 2012; Blandin Knight et al., 2017). However, treatment with traditional platinum-based chemotherapy drugs, such as cisplatin is not considered ideal due to its adverse effects and drug resistance (Siddik, 2003; Addington and Freimer, 2016; Kim, 2016). In recent years, although with the advent of the molecular targeted therapies, it remains a deadly disease with overall 5-year survival rates having not increased significantly over the last 25 years, remaining at approximately 15% (Johnson et al., 2014; Zappa and Mousa, 2016). Accordingly, it is urgent to discover and develop novel agents with low toxicity and effective therapies for the treatment of NSCLC.

**Figure 1:**
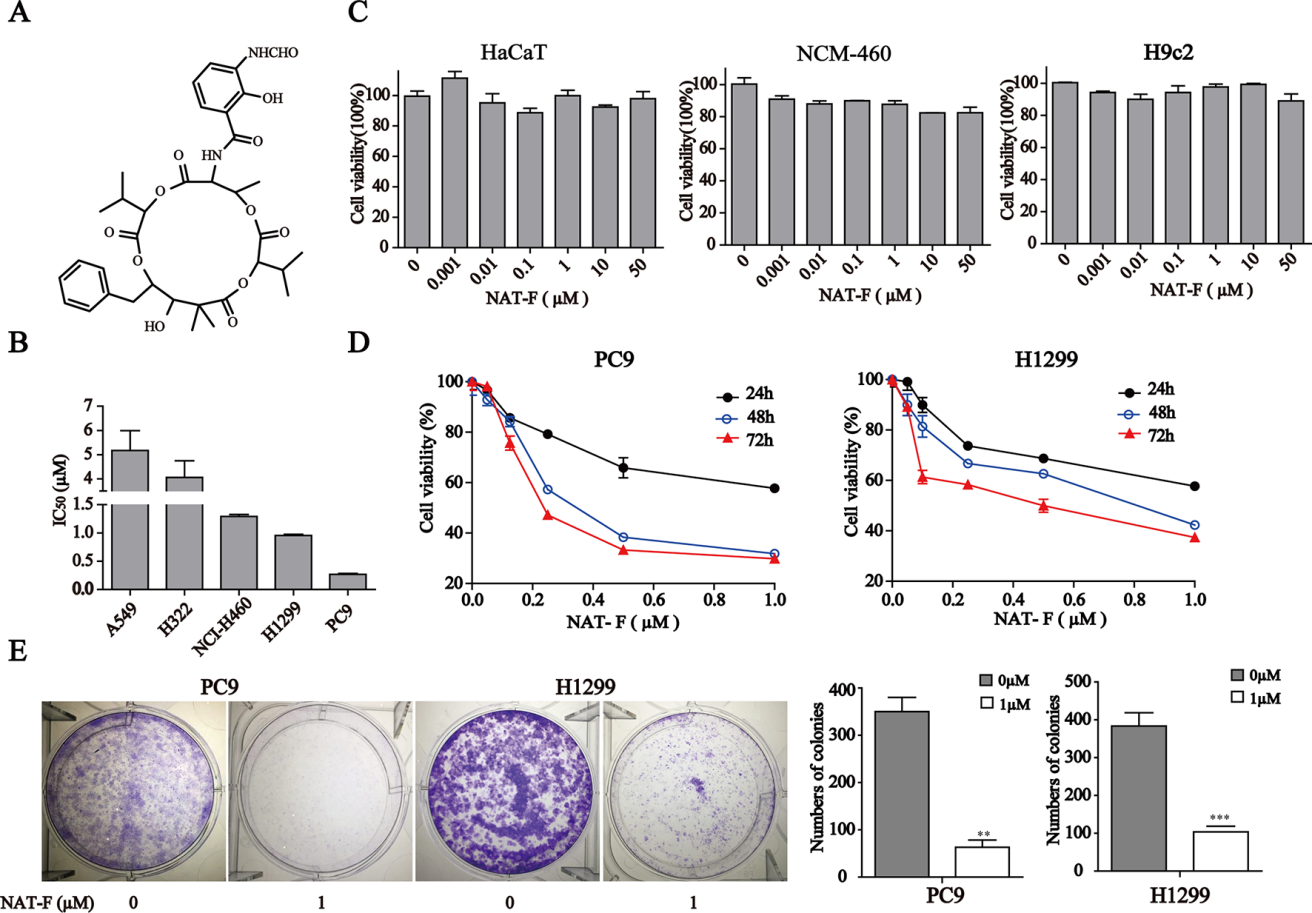
NAT-F inhibited cells proliferation. **(A)** Chemical structure of NAT-F. **(B)** Various types of human NSCLC cells were treated with a concentration range of NAT-F for 48 h. The cell viability was determined using the CCK8-assay. The IC_50_ of each cell line was expressed as the mean ± SD of three independent determinations. **(C)** Toxicity of NAT-F on three normal types cell lines, including NCM-460, HaCaT, and H9c2 cells. Viability was examined by the CCK-8 assay. **(D)** Effect of NAT-F on cell viability of PC9 and H1299. The cells were assayed using the CCK-8 method after treatment with raising doses of NAT-F for 24, 48, and 72 h. Data were expressed as mean ± SD of triplicate experiment. **(E)** NAT-F inhibited the formation of PC9 and H1299 cells colonies. **p < 0.01, ***p < 0.001, significant difference between NAT-F-treated groups and the control.

Natural *Streptomyces*-derived products have received increasing attention owing to their wide variety of biological activities, especially their anti-tumor activities (Calcul et al., 2012; Valipour et al., 2018). In addition, these natural *Streptomyces*-derived products have also been known as resources of clinical anti-tumor drugs, like doxorubicin, rapamycin, mithramycin, and so on (Newman and Cragg, 2007; Olano et al., 2009). The neoantmycins (NATs) are a rare kind of microbial natural products, originally isolated from a South American soil of *Streptomyces orinoci* in 1960s (Caglioti et al., 1969). Neoantimycin F (NAT-F) (**Figure 1A**), a new member of NATs, with its derivatives, including D and E, were previously isolated from *Streptoverticillium orinoci* in 2013, but they were demonstrated to have no significant anti-proliferative activity in HT-29 colorectal cancer cells (Li et al., 2013). Subsequently, it has been reported that NAT-F and its derivatives A, G, and H are exceptionally potent inhibitors of oncogenic K-Ras plasma membrane (PM) localization with low IC_50_ values 3 to 10 nM; they also exhibited cytotoxic activity against the colon cancer cell line SW620 and its daughter cell line SW620 Ad300 with low IC_50_ values 0.04 to 0.61 µM (Salim et al., 2014). Besides, Lim et al. reported that unantimycin A and SW-163A, analogues of neoantimycin, isolated from *Streptomyces* sp. RK88-1355, showed moderate cytotoxicity activities against several cancer cell lines, including HeLa, HL-60, and others; both of them also possessed potent antimalarial activities (Lim et al., 2016). Recently, we studied the biosynthetic pathways of *Streptomyces conglobatus* and isolated a series of NATs, including the known NAT-A, F, H, and a new NAT-I, and revealed that these compounds displayed interesting anti-cancer properties against multiple cell lines (Zhou et al., 2018). In this study, we investigated NAT-F’s growth inhibitory and apoptotic effects on human NSCLC cells and preliminarily elucidated the underlying molecular mechanism.

## Materials and Methods

### Chemicals and Materials

NAT-F was isolated from *Streptomyces conglobatus* by our group (Zhou et al., 2018). NAT-F was dissolved in dimethyl sulfoxide (DMSO) to a concentration of 10 mM DMSO and stored at −20°C. Working solution of NAT-F was diluted in fresh medium to the final concentrations. Primary antibodies of caspase-3 (9662), caspase-9 (20750), Bax (5023), Bcl-2 (15071), Bcl-x_L_ (2762), Mcl-1 (4572), cyclinB1 (4135), cyclinD1 (2978), cyclinE1 (4132), Cdc25A (3652), CDK2 (2546), CDK4 (12790), Chk1 (2360), p-Chk1(S345) (2348), p38 (9212), p-p38 (4511), JNK (9252), p-JNK (9255), ERK1/2 (9102), p-ERK1/2 (4370), γ-H2AX (Ser139) (7631), and GAPDH (5174) were purchased from Cell Signaling Technology (Danvers, MA, USA). Antibodies against cytochrome c (133504) and 8-OHdG (62623), were purchased from (Abcam, United Kingdom).

### Cell Cultures

Human lung cancer cell lines A549 (human lung adenocarcinoma), PC9, H1299, H322 (human bronchoalveolar carcinoma cell lines), NCI-H460 (human large cell carcinoma), and three types of normal cells that included NCM-460 (normal human colon mucosal epithelial cell line), HaCaT (a spontaneously immortalized skin keratinocyte cell line), and H9c2 (embryonic rat heart-derived cells). These cell lines were obtained from the Shanghai Institute of Cell Biology, Chinese Academy of Sciences (Shanghai, China). Cells were cultured in RPMI-1640 (PC9, H1299, H322, and NCI-H460), DMEM/F12K (A549), DMEM (HaCaT, NCM-460, and H9c2) (Gibco, USA) medium supplemented with 10% fetal bovine serum (FBS) (Gibco, USA), 100 U/mL penicillin, and 100 µg/mL streptomycin. All these cells were maintained in a humidified atmosphere with 5% CO_2_ at 37°C.

### Cell Viability Assay

Cell viability was determined by a CCK-8 assay. Briefly, cells were seeded in 96-well plates at a density of 3 × 10^3^ cells/well and grown for 24 h to adhere at 37°C. Then, different concentrations of NAT-F were added to 96-well plates for an indicated time. Ten microliters CCK-8 reagent was added to each well, and the cells were further incubated for 0.5 to 4 h at 37°C. Afterward, the absorbance of each culture cell was measured at 450 nm using a microplate (spectra MAX190; Molecular Devices, USA).

### Cell Cycle Analysis

After treatment with various concentrations (0, 0.03, 0.3, and 1 μM) NAT-F for 24 h, the cells were collected and washed with PBS twice and then fixed with ice-cold 70% ethanol for overnight at 4°C. PI/RNase staining buffer (BD Pharmingen, San Diego, CA, USA) was then added, and the cells were incubated at room temperature (RT) for 15 min in the dark. The cell cycles were analyzed using Attune NxT flow cytometer (Thermo Fisher Scientific, Eugene, Oregon, USA) cell-cycle analysis software.

### Apoptosis Assay Annexin V-PI Staining

Apoptosis analysis was measured using an Annexin V-FITC/PI Apoptosis detection kit (BD Pharmingen, San Diego, CA, USA) according to the manufacturer’s introduction. PC9 and H1299 cells were plated in six-well plates (3 × 10^5^ cells/well) and allowed to attach for 24 h. The cells were treated with desired concentration NAT-F for 48 h. Then, the cells were harvested, washed with ice-cold PBS twice, and resuspended the 1× binding buffer containing 5 μL Annexin V-FITC and 5 μL PI for 15 min at RT in the dark. After labeling, a 400 μL × binding buffer was added into each sample before analysis using flow cytometry (Thermo Fisher Scientific, Eugene, Oregon, USA). At least 10,000 cells were analyzed for each sample. Early apoptotic cells will be positive for Annexin V-FITC (green fluorescence) but negative for PI staining, whereas in late apoptosis will be positive for both Annexin V-FITC and PI staining (red and green fluorescence, respectively).

### 4,6-Diamidino-2-Phenylindole Dihydrochloride Staining

4,6-diamidino-2-phenylindole dihydrochloride (DAPI) staining was used to analyze the cell nuclear morphology. PC9 and H1299 cells were treated with NAT-F for the indicated concentration and time courses. The cells were washed with PBS and fixed with 4% paraformaldehyde for 30 min at RT. After washing with PBS, the cells were stained with DAPI (100 ng/ml) for 15 min in the dark and photographed using a fluorescence microscope (Nikon, Japan).

### Western Blotting

For the extraction of total protein, the cells were harvested, lysed with RIPA buffer (Beyotime Biotechnology, Beijing, China), and cell lysates were centrifuged for 30 min (12,000*g*, 4°C). The supernatant was collected, and the total proteins were quantified using a BCA protein assay kit (Beyotime Biotechnology). The extraction of cytosolic proteins was performed by using Minute^TM^ cytoplasmic fractionation kit according to the manufacturer’s instructions (Invent Biotechnologies, USA). Equivalent amounts of protein were loaded and separated by 10% to 15% sodium dodecyl sulfate-polyacrylamide gel electrophoresis (SDS-PAGE) and then transferred to polyvinylidene ﬂuoride (PVDF) membrane. The membranes were blocked with blocking solution for 1.5 h at RT and then incubated with specific primary antibody at 4°C overnight. Next day, the membranes were repeatedly washed with 0.1% Tris-buffered saline-Tween-20 (TBST), the blots were then incubated with HRP-conjugated secondary antibody (1:3000 dilution; Cell Signaling Technology, Inc., Danvers, MA, USA) in blocking solution for 1 h at RT. After washing with TBST buffer for three times, the protein bands were detected by mixing equal parts of the Stable Peroxide Solution and the Luminol/Enhancer Solution (34077; Thermo Fisher Scientific, USA) and profiled by Amersham Imager 600 gel imaging system (GE Healthcare, USA).

### Measurement of Intracellular ROS Generation

ROS determination was performed by using dichlorofluorescein diacetate (DCFH-DA; Sigma-Aldrich, USA). H1299 and PC9 cells were seeded in six-well plates (3 × 10^5^ cells/well). The cells were treated with 0, 0.03, 0.3, and 1 μM of NAT-F for 24 h. Then, the cells were incubated with DCFH-DA (10 μM, 30 min, in the dark at 37°C) in DMEM medium. After being washed with PBS for twice, the ﬂuorescent signal of the cells was analyzed by using ﬂow cytometer FACS Verse (Thermo Fisher Scientific, Eugene, Oregon, USA) or an inverted microscope system (Nikon, Japan).

### Mitochondrial Membrane Potential (MMP) Determination

The cells were incubated with 100nM tetramethylrhodamine methyl ester (TMRM) (Invitrogen, USA), a cationic fluorescent dye which is widely used to assess the changes in ∆Ψm and mitochondrial permeability transition, for 30 min at 37°C in PBS. After incubation, the cells were washed twice with PBS and then collected for observation with an inverted microscope system or analysis by flow cytometry, respectively.

### Statistical Analysis

All data were expressed as the mean ± standard deviations (SD) from at least three independent experiments and analyzed with SPSS 17.0 software (Chicago, IL, USA) and GraphPad Prism^®^ 5.0 software. Statistical analyses performed between two groups were analyzed by *t*-test. For comparison of more than two groups, one-way analysis of variance (ANOVA) with Least Significance Difference (LSD)'s multiple-comparison *post hoc* tests were used. *p* < 0.05 was considered statistically significant.

## Results

### NAT-F Inhibited Cell Viability and Cell Proliferation in NSCLC Cells

To evaluate the effect of NAT-F on cell viability, five kinds of human NSCLC cell lines, including A549, H322, NCI-H460, H1299, and PC9, were treated with various concentrations of NAT-F for 48 h, and the cell viability was assessed. As shown in **Figure 1B** and **Supplementary Figure 1A**, the IC_50_ at 48 h were, respectively, 5.18 ± 0.47 µM (A549), 4.06 ± 0.39 µM (H322), 1.25 ± 0.03 µM (NCI-H460), 0.9 ± 0.02 µM (H1299), and 0.25 ± 0.01 µM (PC9). We also used three types of normal cells that included HaCaT, NCM-460, and H9c2 to further investigate the cytotoxicity. Our results showed that NAT-F inhibited the cell viability of HaCaT, NCM-460, and H9c2 cells with their IC_50_ values >50 µM (**Figure 1C**). Considering PC9 and H1299 were more sensitive to NAT-F than other three lung cancer cells, the two cell lines were selected to elucidate the molecular mechanisms of NAT-F’s anti-proliferative effects. NAT-F exhibited a significant dose- and time-dependent inhibition of cell growth in PC9 and H1299 cells (**Figure 1D**). We next investigated whether NAT-F suppressed cell proliferation in the two cell lines using trypan blue assay. Our results showed that NAT-F treatment markedly inhibited the cell proliferation in PC9 and H1299 cells in a time-dependent manner (**Supplementary Figure 1B**). Additionally, the colony formation assay further confirmed that NAT-F significantly decreased the proliferation of PC9 and H1299 cells (**Figure 1E**). Consistently, these results indicated that NAT-F exhibited potent anti-proliferative effect against human NSCLC cells but low cytotoxicity to normal cells.

### NAT-F Arrested the Cell Cycle Progression of NSCLC Cells

**Figure 2:**
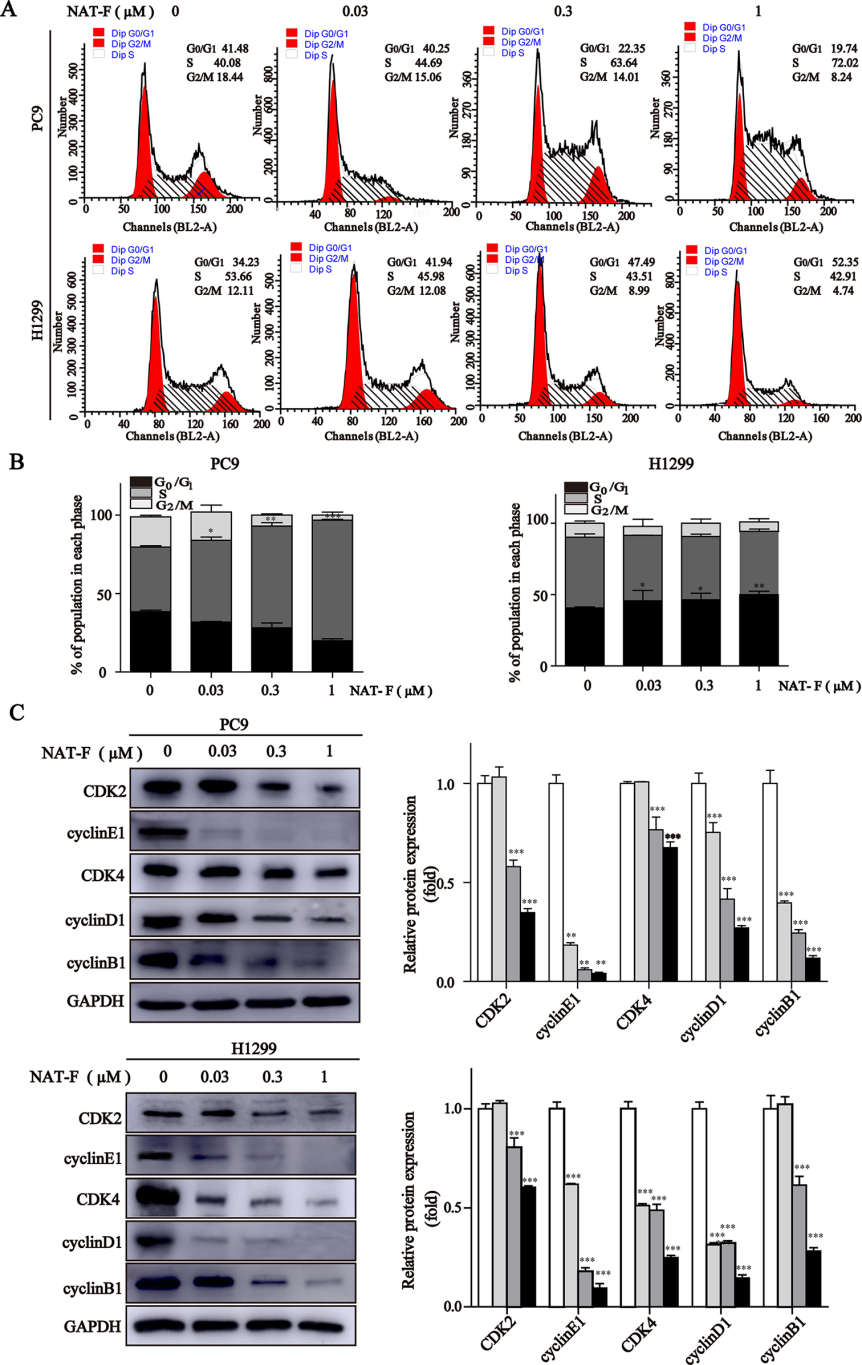
NAT-F induced cell cycle arrest at S or G_0_/_1_ phase in NSCLC cells. **(A)** Cell cycle distribution of PC9 and H1299 cells treated with various concentrations (0, 0.03, 0.3, and 1 μM) of NAT-F for 24 h followed by flow cytometric assay. **(B)** The calculated percentage of cell cycle distribution was showed as mean ± SD from three independent experiments. **(C)** Western blotting of cell cycle phase associated proteins: CDK2, CDK4, cyclinE1, cyclinD1 and cyclinB1. In PC9 cells, samples of CDK2, CDK4, cyclinB1, and GAPDH were from the same gels; samples of cyclinE1 and cyclinD1 were from the same gels. In H1299 cells, samples of CDK2, cyclinE1, cyclinB1, and GAPDH were from the same gels; samples of CDK4 and cyclinD1 were from the same gels. GAPDH was used as internal control. Western blot assay was carried out at least three times, and data represented mean ± SD. *p < 0.05, **p < 0.01, ***p < 0.001, significant difference between NAT-F-treated groups and control.

To further explore whether NAT-F-induced growth inhibition was associated with cell cycle regulation, we assessed the cell cycle distribution by flow cytometry and the expression of cell cycle-regulated proteins by Western blotting subsequently. In PC9 cells, compared with the control, NAT-F mainly induced cell cycle arrest at S phase and led to the corresponding decrease in G_0_/G_1_ and G_2_/M phase in a dose-dependent manner (**Figures 2A, B**). However, in NAT-F–treated H1299 cells, there was a dose-dependent increased cell percentage at G_0_/G_1_ phase, along with a concomitant decrease in S and G_2_/M phases (**Figures 2A, B**). In addition, treatment with different concentrations of NAT-F had no obvious effect on cell cycle in HaCaT cells (**Supplementary Figures 2A, B**).

Next, we measured the expression levels of cell cycle-regulated proteins, such as cyclins and cyclin-dependent kinases (CDKs) in different phases and checkpoint response. As shown in **Figure 2C**, in both PC9 and H1299 cells, NAT-F significantly reduced the expression of cyclin E1 and CDK2, which are known to be involved in G_0_/G_1_ and G_1_/S checkpoint. In NAT-F–treated H1299 cells, we also observed considerable reduction in expression of cyclinD1 and CDK4 that were regarded as central mediators of the G_0_/G_1_ phase transition. However, in PC9 cells, after NAT-F treatment, there was a modest down-regulation in cyclinD1 and CDK4 protein level. Besides, compared with control, the expression level of cyclin B1 was significantly down-regulated in a dose-dependent manner with NAT-F treatment in PC9 and H1299 cells (**Figure 2C**). In summary, the above results clearly indicated that NAT-F may induce cell cycle arrest by controlling the expression levels of key proteins involved in the regulation of different phases in NSCLC cells.

### NAT-F Induced DNA Damage and Apoptosis in NSCLC Cells

**Figure 3:**
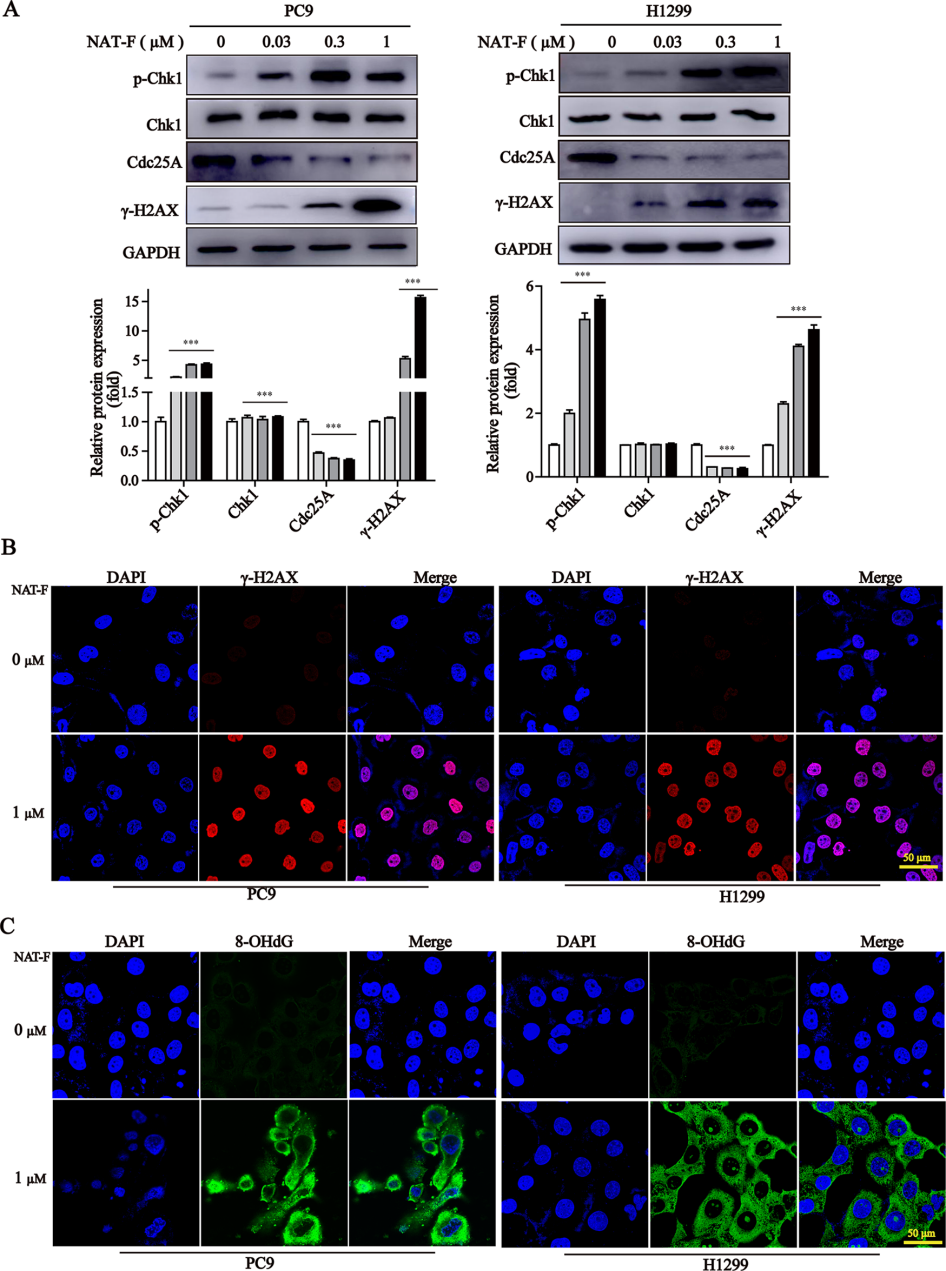
NAT-F induced DNA damage in NSCLC cells. **(A)** The DNA damage regulatory proteins in PC9 and H1299 cells were detected by Western blotting. In PC9 cells, samples of p-Chk1, Chk1, and Cdc25A were from the same gels; samples of GAPDH and γ-H2AX were from the same gels. In H1299 cells, samples of p-Chk1, Chk1, Cdc25A, and GAPDH were from the same gels; samples of γ-H2AX were from the same gels. GAPDH was used as internal control. Western blot assay was carried out at least 3 times, and data represented mean ± SD. Significant differences were indicated by ***p < 0.001 versus the control. **(B)** Immunoﬂuorescence images (630× magnification) of γ-H2AX in PC9 and H1299 cells treated with or without NAT-F(1 μM) treatments for 36 h, respectively. The red ﬂuorescence indicated γ-H2AX foci and the blue ﬂuorescence indicated cell nuclei, respectively. Bar: 50 μm. **(C)** Immunostaining of 8-OHdG in PC9 and H1299 cells with or without NAT-F(1 μM) treatments for 36 h. Representative images (630×magnification) of DAPI (blue color), 8-OHdG staining (green color), and merged were shown for each group of cells. Bar: 50 μm.

**Figure 4:**
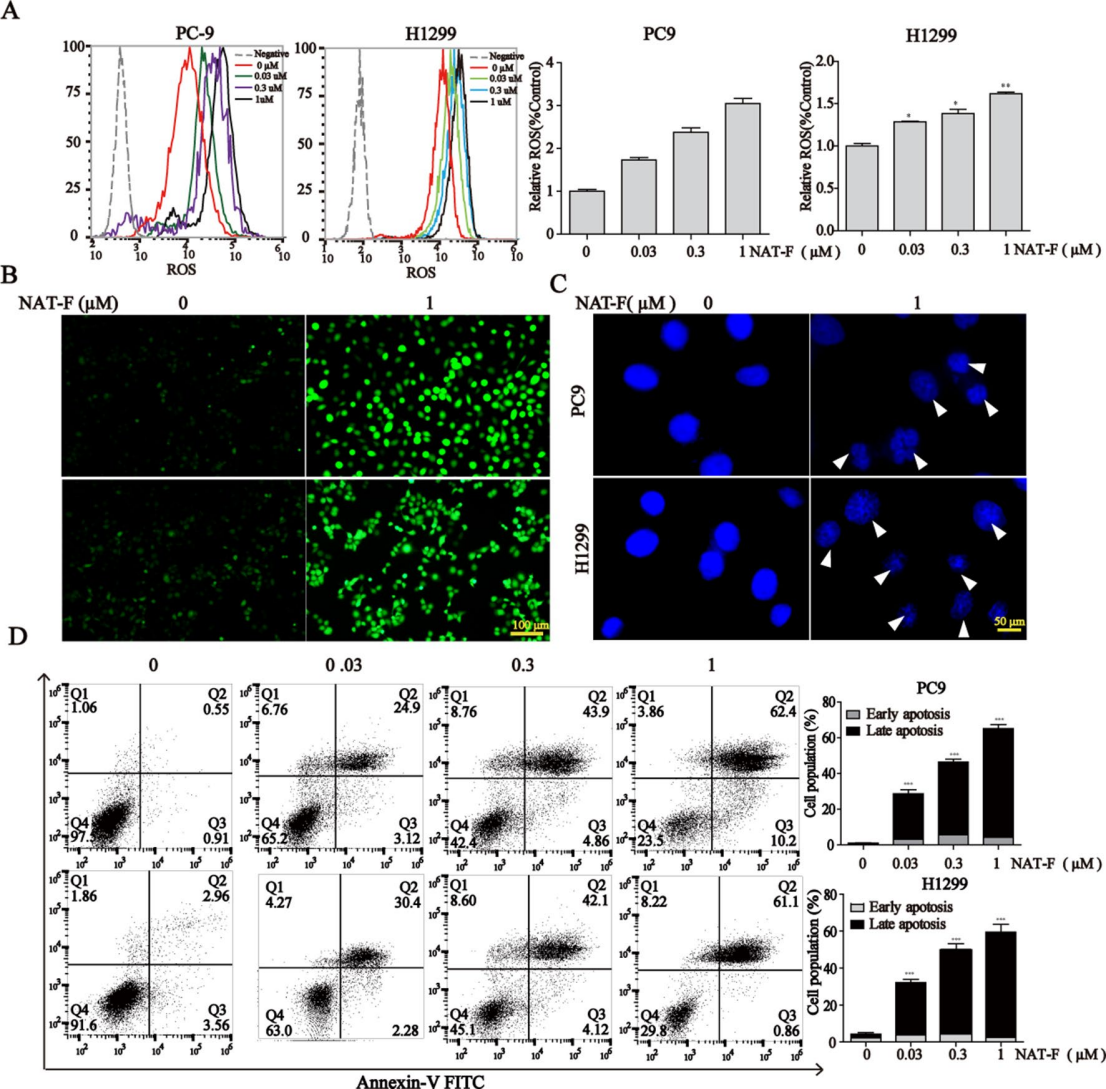
NAT-F–induced ROS overproduction and cell apoptosis in NSCLC cells. **(A)** Cells were treated with various concentrations (0, 0.03, 0.3, and 1 μM) of NAT-F for 24 h. Histograms of the DCFH-DA-stained cells by flow cytometry. Bar graph of the intracellular ROS changes in PC9 and H1299 cells. **(B)** The fluorescence intensity in the two cell lines was observed under fluorescent microscopy. Bar: 100μm. **(C)** The cells were treated with NAT-F for 48 h, stained with DAPI, and then detected by fluorescent microscopy. Representative photographs of PC9 and H1299 cells stained with DAPI staining. Apoptotic cells were characterized with condensed or fragmented nuclei (indicated by white arrows). Bar: 50μm. **(D)** The cells were treated with NAT-F for 48 h, and then they were harvested for Annex-V/PI double staining and measured apoptotic cell death by flow cytometry assay. The total percentage of apoptotic cells represent the average values generated following aggregation of both early and late apoptosis rates. Data represented mean ± SD. *p < 0.05, **p < 0.01 ***p < 0.001, significant difference between NAT-F-treated groups and the control.

It was established that DNA damage could cause cell phase arrest and result in DNA damage repair response (Zhou and Elledge, 2000). As a typical indicator of the amount of DNA damage, the level of γ-H2AX was examined in both NAT-F-treated lung cancer cells. The results suggested that the expression of γ-H2AX was increased in PC9 and H1299 cells after treatment of NAT-F, analyzed by Western blotting and immunofluorescence assay (**Figures 3A, B**). However, there was no significant effect on γ-H2AX levels in NAT-F–treated HaCaT cells (**Supplementary Figure 2C**). Moreover, we further examined the expression of 8-OHdG, which is a valuable biomarker for endogenous oxidative damage to DNA. The immunostaining of 8-OHdG in the lung cancer cells showed that NAT-F treatment significantly increased the levels of 8-OHdG compared with the control group (**Figure 3C**). Induction of ROS production and accumulation elicits oxidative stress that causes prominent damage to DNA. We, therefore, measured ROS production in the two cell lines by flow cytometry or fluorescence microscopy. As shown in **Figures 4A and B**, after treatment with different concentrations of NAT-F, the ROS production was increased, compared with the control group in both PC9 and H1299 cells. Then, we further examined the DNA damage-related signal pathway. As shown in **Figure 3A**, in PC9 and H1299 cells, no significant changes were found in total Chk1 protein, whereas the levels of phosphorylated Chk1 (S345) were obviously increased, and the protein levels of Cdc25A were obviously decreased in a concentration-dependent manner, which indicated that NAT-F–induced DNA damage activated Chk1/Cdc25A signaling pathway.

When DNA damage is beyond the capacity of DNA repair, it eventually results in cell apoptosis. DAPI staining exhibited dramatic differences in nuclear morphology, nuclear shrinkage, DNA fragments, as well as chromatin condensation in NAT-F–treated PC9 and H1299 cells (**Figure 4C**). Moreover, NAT-F significantly induced cell apoptosis or cell necrosis in the two cell lines, as evidenced by Annexin V-FITC/PI double staining by flow cytometric analysis (**Figure 4D**). However, no significant changes were found in NAT-F–treated HaCaT cells (**Supplementary Figure 2D**). Collectively, these results showed that NAT-F could induce severe DNA damage and apoptosis in NSCLC cells.

### NAT-F Triggered Loss of MMP, Mitochondrial Damage, and Activation of Caspases

**Figure 5:**
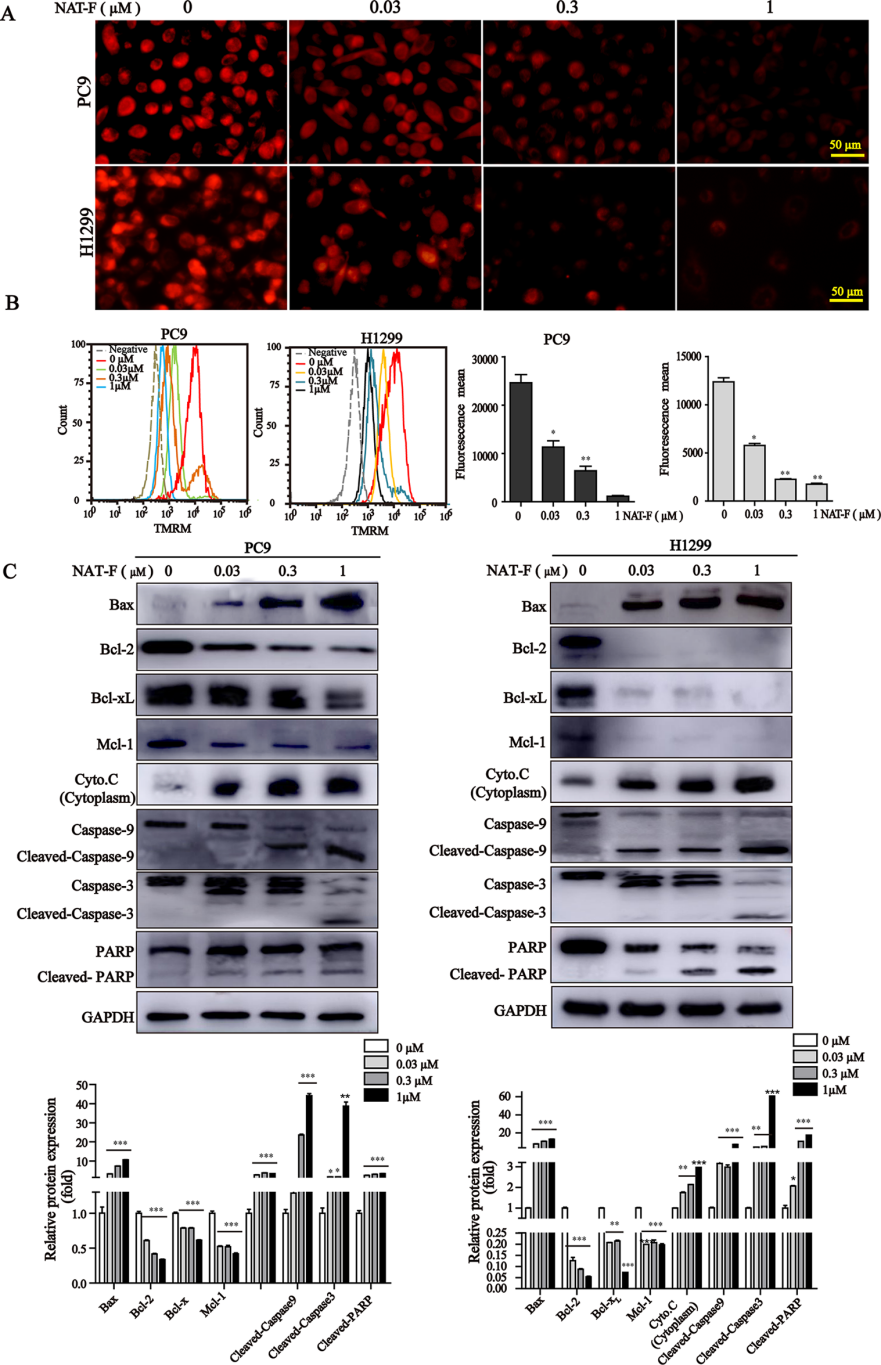
Effect of NAT-F on MMP and apoptosis-related proteins in NSCLC cells *in vitro*. **(A)** Treated cells were stained with TMRM solution and then observed under a fluorescence microscope. Bar: 50 μm. **(B)** Histograms of the TMRM-stained cells by flow cytometry and bar graph of the MMP changes in NAT-F–treated PC9 and H1299 cells. **(C)** Western blot analysis of Bcl-2 family members (Bax, Bcl-2, Bcl-x_L_, and Mcl-1), cytochrome c, cleaved-caspase-9/-3 and cleaved-PARP expressions in PC9 and H1299 cells, and expression level were quantified. In PC9 cells, samples of cleaved-PARP, GAPDH, Bax, Bcl-x_L_, and Mcl-1 were from the same gels; samples of Bcl-2, cleaved caspase-9/-3, cytochrome c (cytoplasm) were from the same gels. In H1299 cells, the gels of these protein samples were similar to that of PC9 cells. GAPDH was used as internal control. Western blot assay was carried out at least three times, and data represented mean ± SD. *p < 0.05, **p < 0.01, ***p < 0.001, significant difference between NAT-F-treated groups and the control.

Loss of MMP is considered as a hallmark of apoptosis typography.There was a significant decrease of MMP in concentration-dependent manner in both lung cancer cells (**Figure 5A** and **Supplemental Figure 3**), and which was further confirmed by TMRM analysis of flow cytometry (**Figure 5B**). Furthermore, to examine the apoptotic pathways activated by NAT-F, we tested the expression of Bcl-2 family members, including Bax, Bcl-2, Bcl-x_L_, and Mcl-1. Western blot analysis indicated that the protein level of pro-apoptotic protein Bax was increased in a concentration-dependent manner after NAT-F treatment in both cell types, whereas the levels of anti-apoptotic protein expression (Bcl-2, Bcl-x_L_, and Mcl-1) were inhibited in a concentration-dependent manner in response to NAT-F treatment (**Figure 5C**). In addition, treatment with NAT-F increased the expression of cytochrome c in the cytoplasm, which was a key event in mitochondria-mediated apoptosis pathway (**Figure 5C**). These results indicated that mitochondrial dysfunction contributed to NAT-F–induced lung cancer cells apoptosis. To further elucidate the mechanism of NAT-F–induced apoptosis, caspases in the mitochondrial apoptotic pathway were examined. As displayed in **Figure 5C**, NAT-F induced a concentration-dependent increase in the expression levels of cleavage caspase-9 and -3 in PC9 cells and similarly increased the expression of these protein in a dose-dependent manner in H1299 cells. Moreover, in cleaved PARP, as another hallmark of apoptosis, NAT-F pre-treatment resulted in an obvious increase of cleavage PARP in a dose-dependent manner in both cell lines (**Figure 5C**). Taken together, the data above showed that intrinsic mitochondrial pathways involved in NAT-F–induced apoptosis in NSCLC cells.

### NAT-F Activated the MAPK Signaling Pathway

**Figure 6:**
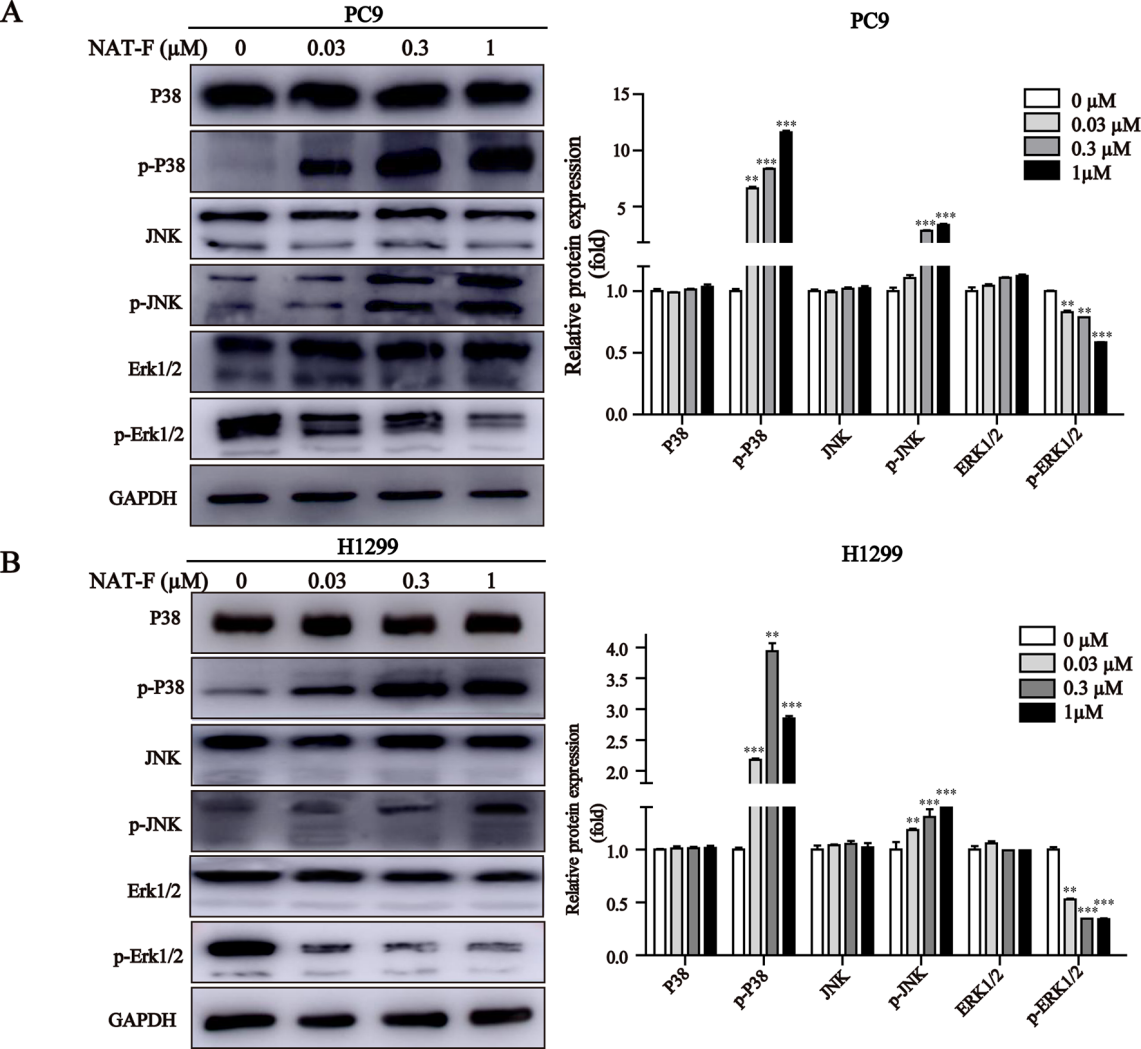
Effect of NAT-F on MAPKs signaling pathway in NSCLC cells. **(A)** and **(B)** Cells exposed to varying concentrations of NAT-F (0, 0.03, 0.3, and 1 μM) were detected for the expression of MAPKs proteins. The protein expressions of p-p38, p38, p-JNK, JNK, p-ERK, and ERK in PC9 and H1299 cells were measured by Western blotting. In PC9 cells, samples of and P38, p-P38, and JNK were from the same gels; samples of p-JNK, ERK1/2, p-ERK1/2, and GAPDH were from the same gels. In H1299 cells, the gels of these protein samples were similar to that of PC9 cells. GAPDH was used as internal control. Quantitative data for MAPKs proteins were shown. Western blot assay was carried out at least three times, and data were expressed as means ± SD. **p < 0.01, ***p < 0.001, significant difference between NAT-F-treated groups and the control.

MAPK signaling pathway plays a crucial role in the control of cellular differentiation, proliferation, oxidative stress, and cell apoptosis. We determined the effects of NAT-F on p38, JNK, and ERK, the three main proteins of MAPK family in NSCLC cells. Our results showed that NAT-F enhanced phosphorylation of p38 and phosphorylated JNK in a concentration-dependent manner, whereas there was no difference in the total protein levels of p38 and JNK (**Figures 6A, B**). However, phosphorylated -ERK1/2 were decreased, compared with the control group (**Figures 6A, B**). These data indicated that NAT-F exerts its apoptotic effects on human NSCLC cells *via* the activation of MAPK signaling pathway.

## Discussion

In this study, we found that NAT-F, a natural *Streptomyces*-derived product isolated from *Streptomyces conglobatus* by our group, displayed anti-proliferative activity in five types of human NSCLC cells and was more potent against PC9 and H1299 cells. In addition, NAT-F was more toxic to human NSCLC cells than to the three types of normal cells. The possible mechanisms of NAT-F’s anti-cancer activities involved in human NSCLC cells were elucidated in our research.

To date, 13 NAT derivatives and their analogs have been isolated since the 1960s, which exhibit various biological activities of immunosuppression, anti-microbial, fungicidal, and anti-tumor effects (Li et al., 2013; Salim et al., 2014; Lim et al., 2016; Zhou et al., 2018). For example, it has been reported that the NAT derivatives SW-163A and B, isolated from the culture broth of *Streptomyces* sp. SNA15896, exerted immunosuppressive and anti-microbial activities *in vitro* (Takahashi et al., 2001). Besides, Izumikawa et al. demonstrated that NAT derivatives JBIR-04 and -05, isolated from *Streptomyces violaceoniger* 4541-SVS3, inhibited the expression of GRP78 induced by 2-deoxyglucose in human fibrosarcoma HT1080 cells (Izumikawa et al., 2007). In the present study, we found that NAT-F potently inhibited the viability and survival of NSCLC cells, and it was an effective anti-tumor agent *in vitro*.

Cell cycle deregulation is one of the major hallmarks of cancer progress, and induction cell cycle arrest is an important cause to inhibit cancer growth and proliferation (Hsiao et al., 2012). NAT-F showed different effect on cell cycle arrest, induced S stage arrest in PC9 cells, but G_0_/G_1_ stage arrest in H1299 cells, which suggested that NAT-F is a cell cycle non-specific anti-tumor compound. We hypothesize that the differences in the effect of NAT-F on cell cycle arrest are primarily determined by the genetics of the cell lines. Indeed, the NSCLC cells used in our study have different TP53 genotype (**Supplementary Table 1**), as reported in the literature (Mukhopadhyay and Roth, 1993; Deng et al., 2007; Tyagi et al., 2013; Pellerano et al., 2019). For example, the human lung cancer cell line PC9 harbors mutant TP53, whereas the cell line H1299 is a TP53-null lung cancer model (Yonesaka et al., 2006; Allen et al., 2015; Song et al., 2019). Nevertheless, NAT-F caused G_1_/G_0_ arrest and apoptosis accompanied by down-regulation of cyclinD1 and cyclinE1 in H1299 (p53-null) cells, indicating that it exerted these effects in a p53-independent manner. Many anti-tumor compounds have different effect on cell cycle, although the underlying mechanism is unclear. Previous work from our laboratory also demonstrated aaptamine, isolated from the marine sponge *Aaptos*, showed different effects on cell cycle in two hepatocellular carcinoma (HCC) cell lines (Li et al., 2015). Natural compounds, such as cephalochromin and xanthoxyletin, have been reported arrested cell cycle at G_0_/G1 or S phase, respectively (Rasul et al., 2011; Hsiao et al., 2014). The exact mechanisms underlying the different cell arrest caused by NAT-F remain unknown and also need to be explored in the future.

The DNA damage response comprises DNA repair, cell cycle checkpoint control, and DNA damage-induced apoptosis, which plays critical roles in promoting genomic integrity and suppresses tumorigenesis (Foster et al., 2012). Histone H2AX phosphorylated on serine 139 (γ-H2AX), an important molecular marker of DNA damage, is widely used to evaluate the DNA double-strand breaks (Bonner et al., 2008; Yang et al., 2018). The oxidative DNA product, 8-OHdG, is a commonly used biomarker for the measurement of endogenous oxidative DNA damage (Valavanidis et al., 2009). It was established that ROS can produce oxidative stress and cause damage to DNA (Klaunig et al., 2010). We also found out that NAT-F significantly induced an increase in oxidative DNA damage, as indicated by the formation of γ-H2AX and 8-OHdG, and these events are correlated with the increase in the production of ROS. Notably, a strong cytoplasmic staining for 8-OHdG was observed in NAT-F–treated cells. 8-OHdG, a form of guanine oxidized at the C-8 position, is a major product of nucleic acid oxidation (Floyd et al., 1988; Lood et al., 2016). In addition, unlike any other species that contains oxidized guanine, 8-OHdG is membrane permeable (Cooke et al.,2005). Moreover, it has been reported that there were abundant cytoplasmic 8-OHdG staining not only in cancer cells but also in tumor tissues (Cao et al., 2006; Sheridan et al., 2009; Tempka et al., 2018). Hence, it can be speculated that 8-OHdG might not be completely distributed in the nucleus. Another possible reason for the observed strong cytoplasmic staining of 8-OHdG is likely due to DNA damage caused by NAT-F, which occurred in both the nucleus and mitochondria. A study by Valavanidis et al. showed that 8-OHdG, one of the main forms of oxidative damage induced by free radicals, was found in nuclear and mitochondrial DNA (Valavanidis et al., 2009). DNA damage at G_1_ or S phase results in cell cycle arrest brought about by Chk1-mediated deactivation of the Cdc25A phosphatase (Zhang et al., 2006). In our study, we found that levels of phosphorylated Chk1 were obviously increased, and Cdc25A was significantly decreased in NAT-F-treated lung cancer cells. We demonstrated that Chk1/Cdc25A pathway is involved in the regulation of DNA damage response in lung cancer cells exposed to NAT-F.

Induction of apoptosis of cancer cells is one of the major mechanisms of anti-tumor agents which play anti-tumor effect (Hsiao et al., 2014). Here, DAPI staining indicated that NAT-F could induce the formation of apoptotic nuclei in PC9 and H1299 NSCLC cells, which was further supported by the result of flow cytometry with Annexin-V/PI staining assay. Apoptosis is known to be stimulated through the extrinsic death receptor-mediated and intrinsic mitochondrial-dependent signaling pathway (Robinson et al., 2011). It is well known that mitochondria is not only the important site of respiration but also oxidative phosphorylation in the cell. The loss and disruption of MMP is recognized to be a hallmark of early apoptosis, where release of cytochrome c from the mitochondria is also the initiation of apoptosis (Gottlieb et al., 2003). Accumulated evidence shows that Bcl-2 family proteins, including pro-apoptotic Bax and Bid and anti-apoptotic Bcl-2, Bcl-x_L_, and Mcl-1 proteins, play a pivotal role in regulation of mitochondrial membrane permeability and the mitochondrial apoptotic pathway (Degterev et al., 2003). For example, proapoptotic Bcl-2 family members, Bax and Bak, play a crucial role in the regulation of apoptosis through permeabilization of the mitochondrial outer membrane, which subsequently lead to the release of pro-apoptotic factors, such as cytochrome c (Korsmeyer et al., 2000). Indeed, our results revealed treatment of NSCLC cells with NAT-F resulted in a drop of MMP in a concentration-dependent manner and significantly elevated the expression of cytochrome c in the cytoplasm. Besides, the results from our Western blotting also showed that NAT-F treatment significantly up-regulated Bax and down-regulated Bcl-2, Bcl-x_L_, and Mcl-1 in both NSCLC cells. These results indicated that NAT-F–induced apoptosis was related to mitochondrial damage. In addition, Bax-induced apoptosis is associated with other mitochondrial dysfunctions, such as alterations of the MMP and accumulation of ROS. Moreover, high levels of ROS generation associated with cellular damage and apoptosis (Yang et al., 2010). Many anti-cancer agents exerted anti-tumor activity by ROS-dependent apoptotic signaling (Yoon et al., 2013; Wang et al., 2014; Jiang et al., 2017). We also showed that NAT-F treatment induced concentration-dependent ROS production in NSCLC cells, which indicated that NAT-F–induced apoptotic cell death in human NSCLC cells was associated with the ROS generation. In addition, caspases are a family of cystine proteases that play critical roles in both intrinsic and extrinsic signaling pathways (Degterev et al., 2003). Initiator caspase-9 and effector caspase-3 could be activated due to the alteration of the apoptotic members, such as Bcl-2 family proteins (Ganesan and Colombini, 2010; Cooley-Andrade et al., 2016). Activation of Bax and Bak lead to the loss of MMP and release of cytosolic cytochrome c to interact with caspase 9 to form a multi-protein complex known as “apoptosome”; this further trigger caspase 3 activation and results in apoptosis (Tsujimoto, 1998; Yuan and Akey, 2013). We observed that NAT-F mediated the activation of caspase-9 and -3, and the cleavage of 116-kDa PARP into 89-kDa subunit revealed that mitochondria-mediated caspase cascade pathway was activated in NAT-F-induced apoptosis.

The mitogen-activated protein kinases (MAPKs) superfamily, including ERK1/2, p38, and JNK, play important roles in the regulation of cell differentiation, proliferation, and apoptosis. In general, the activation of ERK is pro-survival but activation of p38 and JNK can result in apoptosis (Schroeter et al., 2003; Junttila et al., 2008; Krishna and Narang, 2008). It has been reported that oxidative stress, elicited by ROS, was one of the major stimuli of the MAPK cascade, which ultimately resulted in cell survival or apoptosis (Satoh et al., 2000). High levels of ROS promoted cell apoptosis by activating MAPK signaling pathway (Beccafico et al., 2015; Liu et al., 2015). The activation of JNK or p38-MAPK pathway, which is related to oxidative stress, has been confirmed to be involved in the process of neuronal cell apoptosis (Choi et al., 2004). In addition, several studies have shown that the activation of p38-MAPK–mediated mitochondria-dependent apoptosis can be induced by exposure to some agents (Kim et al., 2011; Lu et al., 2011). In our study, we had demonstrated that dose-dependent NAT-F treatment induced ROS production and up-regulated p38 and JNK phosphorylation, and ERK1/2 phosphorylated level was significantly suppressed for NAT-F treatment in both NSCLC cells. These results indicated that ROS-mediated signals were critical for downstream MAPK activation-regulated mitochondria-dependent apoptosis pathway in NAT-F-induced NSCLC cells apoptosis.

Taken together, our results demonstrate that the *Streptomyces*-derived compound NAT-F possesses promising inhibitory effects on proliferation through cell cycle arrest and induces DNA damage in human NSCLC *in vitro*. In addition, NAT-F can induce NSCLC cell apoptosis through the mitochondria-dependent apoptosis pathway, where mitochondrial dysfunction (loss of MMP, the releasing of cytochrome c, and regulation of pro-apoptotic protein and anti-apoptotic protein expression), increased PARP cleavage, and activation of caspase-9 and -3 are critical events for apoptosis. Further investigation demonstrate that activation of MAPKs may be one of the contributors in NAT-F-induced apoptosis.

## Author Contributions

LiyL and HZ designed the experiments, carried out the research, analysed the data, and wrote the manuscript. WW, YS, and YW provided technical assistance with several protocols. XL, YS, and YZ isolated NAT-F from *Streptomyces conglobatus* strain. WW, YW, LiL, and JT provided support in maintenance of cell culture and proof read the manuscript. FS and H-WL conceived the study, planned the project, and coordination of the study. All authors read and approved the final manuscript.

## Funding

This research was supported by National Key Research and Development Program of China (2018YFC0310900), National Natural Science Foundation of China, Grant/Award Number: U1605221, 81502936, 31670096, 21502113.

## Conflict of Interest Statement

The authors declare that the research was conducted in the absence of any commercial or financial relationships that could be construed as a potential conflict of interest.
