# Supplementary figures and images for: Neoantimycin F, a Streptomyces-Derived Natural Product Induces Mitochondria-Related Apoptotic Death in Human Non-Small Cell Lung Cancer Cells
Source: Front Pharmacol. 2019 Sep 18;10:1042. doi: 10.3389/fphar.2019.01042 (PMC6760012; doi:10.3389/fphar.2019.01042)

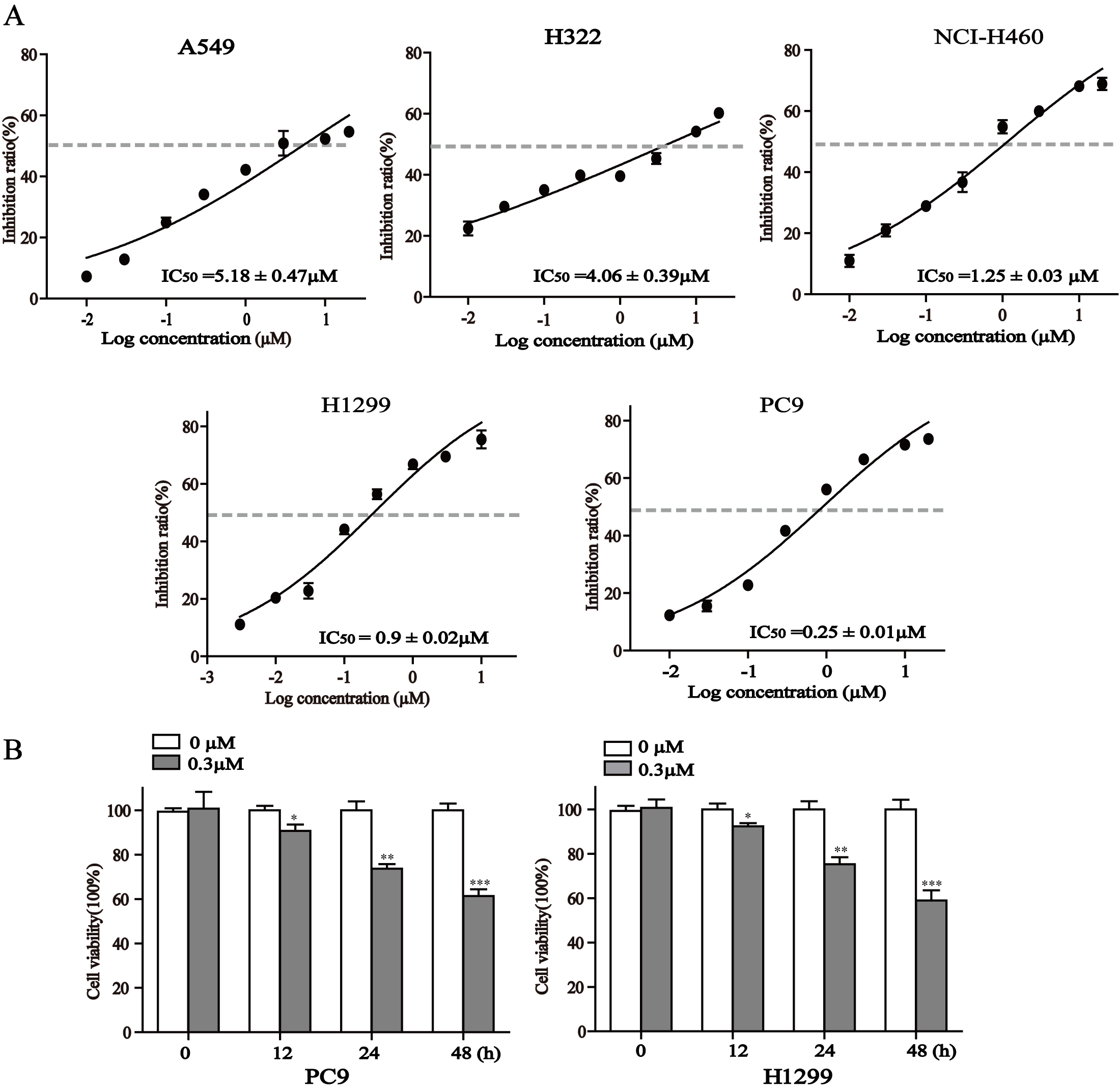

Supplement: Supplementary file 1 [file Image_1.tif]

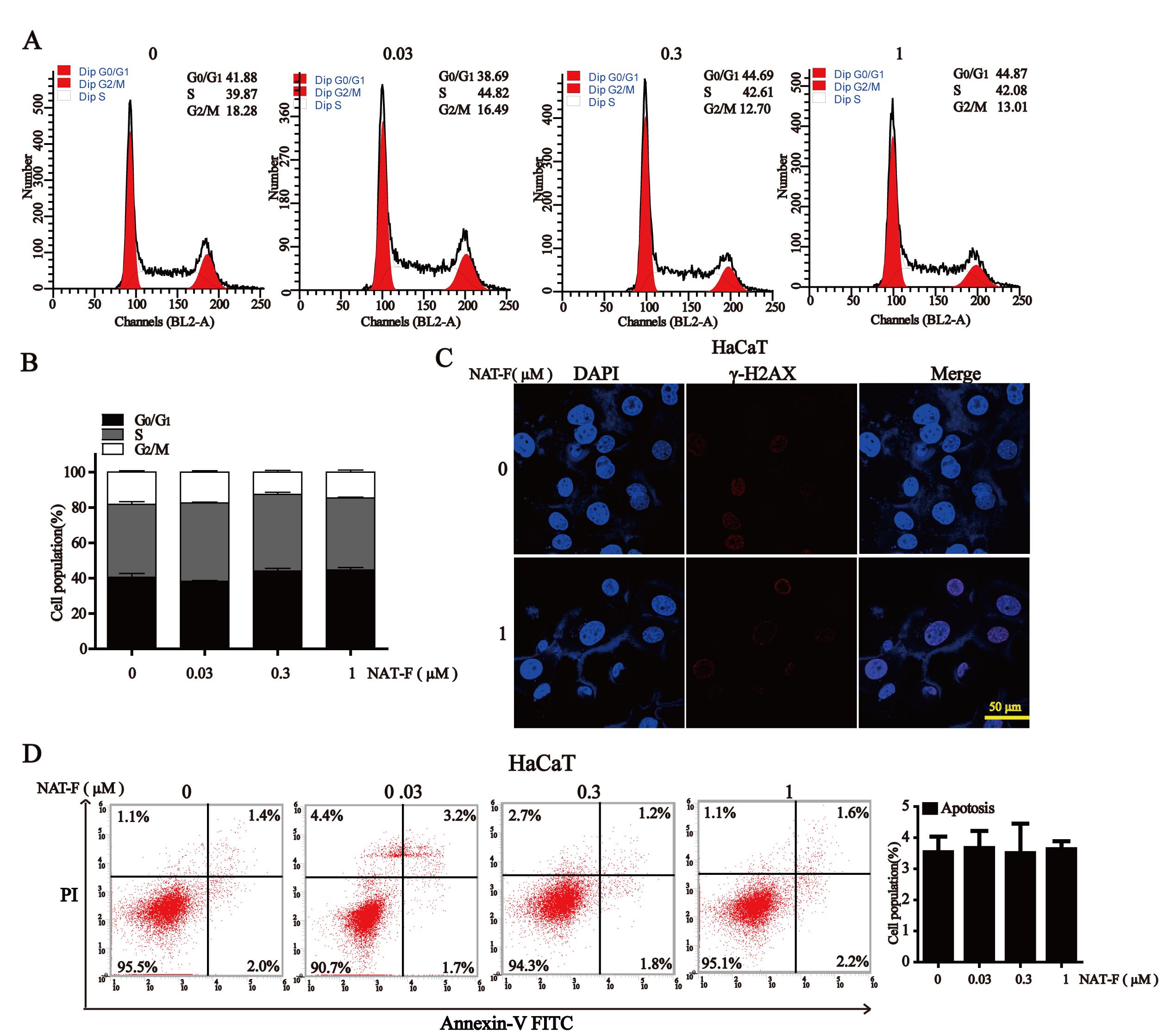

Supplement: Supplementary file 2 [file Image_2.tif]

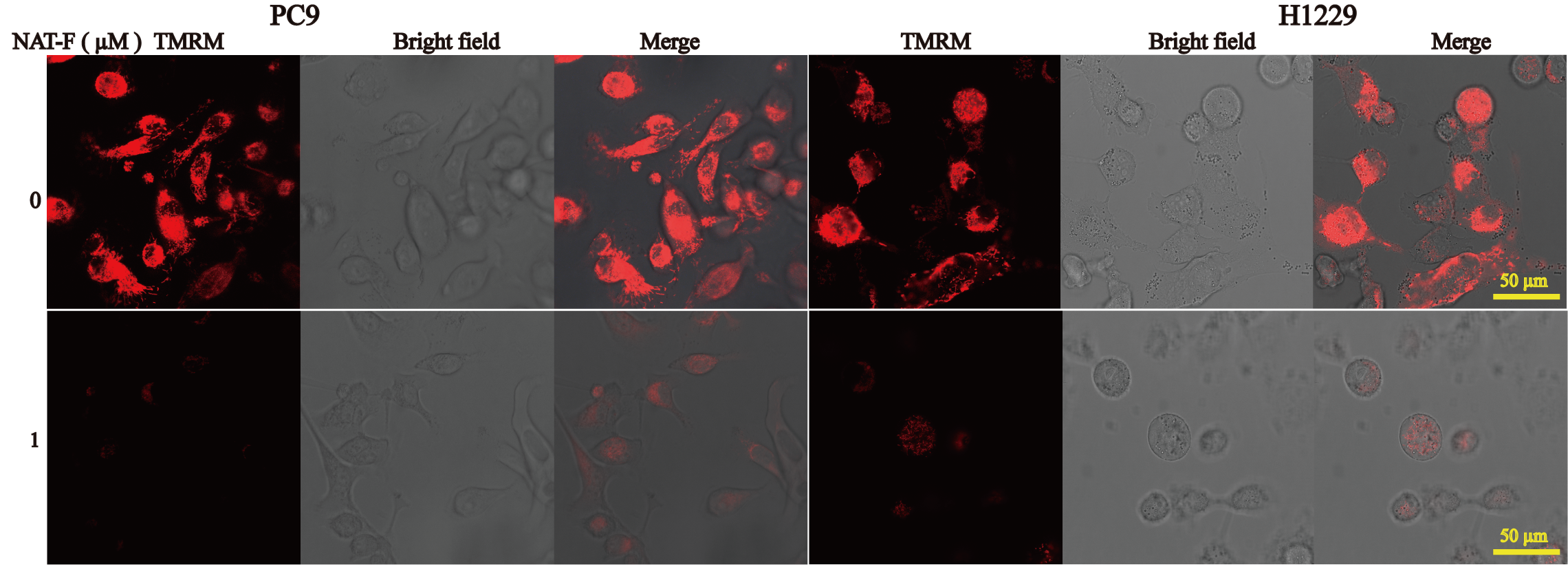

Supplement: Supplementary file 3 [file Image_3.tif]
