# Supplementary material for: Neoantimycin F, a Streptomyces-Derived Natural Product Induces Mitochondria-Related Apoptotic Death in Human Non-Small Cell Lung Cancer Cells
Source: Front Pharmacol. 2019 Sep 18;10:1042. doi: 10.3389/fphar.2019.01042 (PMC6760012; doi:10.3389/fphar.2019.01042)
Supplement: Supplementary file 4 [file Table_1.docx]

SUPPLEMENTARY MATERIAL

**Neoantimycin F，a *Streptomyces*-derived Natural Product Induces Mitochondria-Related** **Apoptotic Death in Human Non-small Cell Lung Cancer Cells**

Liyun Liu^1,#^, Hongrui Zhu^2,#^ , WeiWu^1^, Yaoyao Shen^1^, XiaoLin^3^, Ying Wu^1^, Yongjun Zhou^1^, LiLiu^1^, Jie Tang^1^, Fan Sun^1,*^, Hou-Wen Lin^1,*^

**I.** **Supplementary Methods**

**1. Colony formation assay**

PC9 and H1299 cells (500/well) were seeded in 6-well plates for 24 h and then treated with NAT-F(1μM) at different concentrations for another 24 h. After that, cells were washed with PBS, and cultured in fresh medium for 10 days. Finally, the cells were fixed in 4% [paraformaldehyde](https://www.sciencedirect.com/topics/medicine-and-dentistry/paraformaldehyde) for 30 min and stained with 0.1% crystal violet for 30 min and counted manually.

**2.Trypan Blue Assay**

PC9 and H1299 cells were seeded in 6-well plates and incubated overnight prior to treatment with NAT-F(0.3μM) for various time periods. Then the cells were stained with 0.4% trypan blue solution (Beyotime Biotechnology, Beijing, China) and both live cells (unstained) and dead cells (stained in blue) were counted using a hemocytometer.

Cell vitality were measured under the following formula: Living cell rate (%) = number of living cells / (total number of living cells + dead cells) ×100%

**3.Immunofluorescence**

The cells were fixed in 4% paraformaldehyde for 30 min at 37 °C, and permeabilized with 0.03% Triton X-100 for 10 min. Then the cells were blocked with 3% bovine serum for 30 min, and incubated with primary antibodies overnight at 4 °C (1:1000 for anti-γ-H2AX, 1:500 for 8OH-dG). After three washes with PBS, cells were incubated with secondary antibodies (Life Technologies, Carlsbad, CA). Cells were then washed in PBS and further incubated with 100 ng/mL DAPI (Sigma) for 10 min. Finally, the cells were subjected to laser confocal microscopy (Leica SP8) analysis.

**II. Supplementary Figure Legends**

**Supplementary Figure 1** Anti-proliferative effect of NAT-F in NSCLC cells. **(A)** Dose response curves and corresponding IC_50_ values for five lung cancer cell lines treated with different concentrations of NAT-F for 48h. All experiments were performed in triplicate. **(B)** The effects of NAT-F on NSCLC cells were seeded onto 12 well plates and treated with NAT-F (0.3μM) for various time periods. The live cells and dead cells were counted were evaluated by trypan blue assay. Data are expressed as the mean ± SD of three independent experiments. Significance was determined by the Student’s t-test (^*^*p*<0.05, ^**^*p* < 0.01, ^***^*p* < 0.001 vs. the control).

**Supplementary Figure 2**

Effect of NAT-F on cell cycle, DNA damage and apoptosis in HaCaT cells. (**A**) Cell cycle distribution of HaCaT cells treated with various concentrations (0, 0.03, 0.3, and 1μM) of NAT-F for 24 h followed by flow cytometric assay. (**B**) Calculated the percentage of cell cycle distribution was showed as mean ± SD from three independent experiments. (**C**) Representative NAT-F-induced DNA damage visualized by γ-H2AX labelling under a confocal microscope. Bars: 50 μm.(**D**) Flow cytometric analysis of cell apoptosis with Annexin V-FITC/PI double staining. Quantification of the apoptotic cells in both early and late stage.

**Supplementary Figure 3** Effect of NAT-F on MMP in NSCLC cells. Representative images (630× magnification) of TMRM staining of PC9 and H1299 cells treated with or without NAT-F (1μM) for 48h.Bar: 50μm.

**III.** [**Supplementary Table 1**](https://www.ncbi.nlm.nih.gov/pmc/articles/PMC5108342/#sup1)**.** The status of *p53* genes in human NSCLC cell lines

|  | PC9 | H1299 | A549 | H460 | H322 |
| --- | --- | --- | --- | --- | --- |
| **p53** | Mutant^a^ | Null | Wild-type | Wild-type | Mutant^b^ |

^a^: PC9 cells have a specific isoform of p53 (c.743G>A) and express the mutant p53 R248Q.

^b^: H322 cells have the specific isoform p53 (c.743G>T) and express the mutant p53 R248L.
